# Structural basis for prodrug recognition by the SLC15 family of proton-coupled peptide transporters

**DOI:** 10.1073/pnas.1813715116

**Published:** 2019-01-02

**Authors:** Gurdeep S. Minhas, Simon Newstead

**Affiliations:** ^a^Department of Biochemistry, University of Oxford, OX1 3QU Oxford, United Kingdom

**Keywords:** membrane transport, drug transport, SLC15, proton-coupled transport

## Abstract

A major challenge in drug development is the optimization of intestinal absorption and cellular uptake. A successful strategy has been to develop prodrug molecules, which hijack solute carrier (SLC) transporters for active transport into the body. The proton-coupled oligopeptide transporters, PepT1 and PepT2, have been successfully targeted using this approach. Peptide transporters display a remarkable capacity to recognize a diverse library of di- and tripeptides, making them extremely promiscuous and major contributors to the pharmacokinetic profile of several important drug classes, including beta-lactam antibiotics and antiviral and antineoplastic agents. Of particular interest has been their ability to recognize amino acid and peptide-based prodrug molecules, thereby providing a rational approach to improving drug transport into the body. However, the structural basis for prodrug recognition has remained elusive. Here we present crystal structures of a prokaryotic homolog of the mammalian transporters in complex with the antiviral prodrug valacyclovir and the peptide-based photodynamic therapy agent, 5-aminolevulinic acid. The valacyclovir structure reveals that prodrug recognition is mediated through both the amino acid scaffold and the ester bond, which is commonly used to link drug molecules to the carrier’s physiological ligand, whereas 5-aminolevulinic acid makes far fewer interactions compared with physiological peptides. These structures provide a unique insight into how peptide transporters interact with xenobiotic molecules and provide a template for further prodrug development.

## Significance

Poor oral bioavailability is one of the leading causes of compound failure in drug development and a major challenge for the pharmaceutical industry. A successful approach to address this challenge has been the development of prodrugs that target the intestinal peptide transporter, PepT1 (SLC15A1). PepT1 exhibits a remarkably promiscuous binding site and is known to transport many different drug molecules, making it an excellent target for prodrug design and delivery. However, the structural basis for drug recognition remains largely unknown. Here we present the structure of a bacterial homolog of PepT1 bound to both an antiviral prodrug, valacyclovir, and anticancer drug 5-aminolevulinic acid. These structures enable a pharmacophore model to be developed that will aid future prodrug design.

Solute carrier (SLC) transporters are increasingly being recognized as important determinants of drug efficacy in clinical trials and as important therapeutic targets (1, 2). Poor oral bioavailability is one of the leading causes of compound failure in preclinical and clinical drug development and a major challenge for the pharmaceutical and biotechnology industries (3). A successful approach to address this challenge has been the development of prodrugs that target the intestinal peptide transporter, PepT1 (SLC15A1) (4) (*SI Appendix*, Fig. S1). Prodrugs are bioreversible derivatives of drug molecules that undergo an enzymatic or chemical transformation in vivo to release the active parent drug (5). Over the past 10 years significant effort has been made in the design of novel prodrug molecules with improved pharmacokinetic profiles (6). However, targeting specific SLC transporters for carrier-mediated uptake is still a major challenge. PepT1 exhibits a remarkably promiscuous binding site and is known to transport many different drug molecules. These include, but are not limited to, angiotensin converting enzyme inhibitors, beta-lactam antibiotics, an N-methyl-d-aspartate receptor antagonist PD-15874, and 5-aminolevulinic acid, an endogenous nonprotein amino acid currently being evaluated as a photodynamic therapeutic agent for the treatment of bladder cancer and esophageal carcinoma (7). While PepT1 is the first peptide transporter encountered by drugs following oral dosage, a second peptide transporter, PepT2 (SLC15A2), functions to selectively reuptake peptides in the nephron and also functions in peptide transport across the blood brain barrier (8). As such, prodrugs targeting both PepT1 and PepT2 show favorable absorption and retention profiles in animal models of drug disposition and are being actively pursued as valid targets for improving pharmacokinetic profiles (9).

A major breakthrough in carrier-mediated prodrug development was the introduction of the antiviral valacyclovir, marketed under the trade names Valtrex and Zelitrex. Valacyclovir (Cambridge Chemical Database ID: TXC) is a prodrug derivative of the antiviral agent acyclovir, which is used in the treatment of disease caused by herpes viruses (including herpes zoster, HSV-1 and -2) as well as in prophylaxis against acquisition of infection and in suppression of latent disease (10). The oral bioavailability of valacyclovir improved to >50% for the prodrug derivative valacyclovir compared with 15% for the parent drug acyclovir, which was attributed to its recognition and transport by PepT1 (11, 12). Although the extreme promiscuity displayed by PepT1 has made it a major focus of prodrug strategies (7), the structural basis for prodrug recognition is still enigmatic. Lack of structural information on how prodrugs interact with the transporter is hampering efforts to design accurate pharmacophore models for, among other developments, computer-aided drug design (13).

To date, bacterial peptide transporters have proven to be valid and reliable model systems with which to understand the molecular basis of peptide recognition within the human PepT1 and PepT2 transporters (14, 15). PepT1 and PepT2 belong to the much larger POT or PTR family of proton-coupled oligopeptide transporters, with homologs found in all domains of life except the archaea (16, 17). POT family transporters belong to the major facilitator superfamily (MFS) of secondary active transporters, and use the proton electrochemical gradient to drive the concentrative uptake of di- and tripeptides into the cell (18). Although crystal structures of bacterial POT family transporters have revealed that peptides can be accommodated in distinct binding positions (19, 20) and transported with variable proton stoichiometries (21), the rules governing how specific functional groups on peptides are recognized remain obscure.

To address this question and understand how peptide-based prodrugs and nonproteinogenic drug molecules are recognized and transported via the SLC15 family, we determined the crystal structure of a bacterial POT family member in complex with both valacyclovir and 5-aminolevulinic acid at 3.1-Å and 2.5-Å resolution, respectively. Combined with the previous peptide-bound structures, we present a pharmacophore model for prodrug recognition, which facilitates a structure-based route to further drug development targeted at the SLC15 family.

## Results and Discussion

### Structure of Valacyclovir Complex.

Recently we discovered a prokaryotic homolog of PepT1 from the bacterium *Staphylococcus hominis*, PepT_Sh_ (*SI Appendix*, Fig. S2), which was able to transport a natural thioalcohol-conjugated peptide, exhibiting structural characteristics similar to prodrug molecules (22). We therefore reasoned that PepT_Sh_ may also be able to recognize and transport valacyclovir. Using a competition assay we determined that valacyclovir was able to compete for dipeptide binding in PepT_Sh_ with an IC_50_ value of 7.4 mM (*SI Appendix*, Fig. S3*A*). Although higher than equivalent IC_50_ values obtained for natural peptides, which were 72.2 μM for dialanine (AlaAla) and 23.7 μM for trialanine (AlalAlaAla), similar K_M_ values have been reported for valacyclovir uptake in mouse and human PepT1 (23, 24). We were further able to measure both valacyclovir and 5-aminolevulinic acid transport directly using a pyranine-based transport assay that measures proton movement (21) (*SI Appendix*, Fig. S3 *B* and *C*), supporting the use of PepT_Sh_ as a model for understanding prodrug transport in the mammalian proteins.

**Fig. 1.**
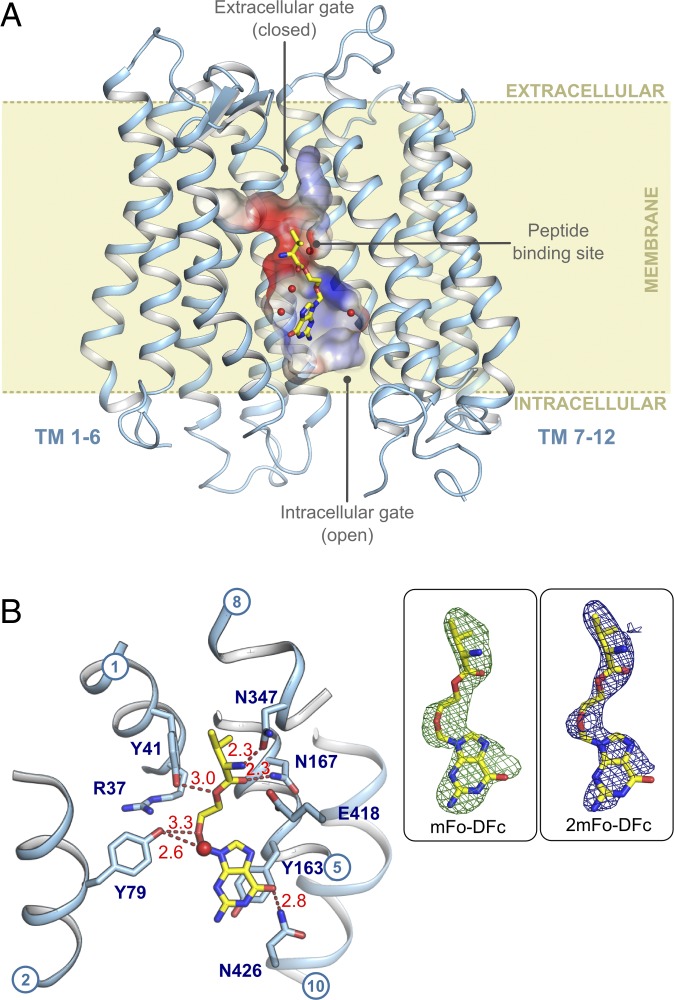
Crystal structure of PepT_Sh_ in complex with valacyclovir. (*A*) PepT_Sh_ represented as light blue helices in the plane of the membrane. The binding cavity surface is colored according to the localized electrostatic potential. Yellow sticks represent the bound prodrug, valacyclovir. Waters are shown as red spheres. (*B*) Residues that interact with valacyclovir are shown as blue sticks. Hydrogen bonds are shown as red dashes and distances are labeled. (*Inset Left*) Experimental *m*Fo-*D*Fc difference electron density (green mesh) for valacylcovir, contoured at 3 σ. (*Inset Right*) Final refined 2*m*Fo-*D*Fc electron density (blue mesh), contoured at 1 σ.

Following extensive screening, a crystal structure of PepT_Sh_ in complex with valacyclovir was subsequently determined using the in meso crystallization method and refined to a final resolution of 3.1 Å (*SI Appendix*, Table S1). PepT_Sh_ adopts an almost identical inward open conformation to that obtained in our previous study (22), with a root mean square deviation (rmsd) of 0.499 Å for 480 C_α_ atoms (Fig. 1*A*). The valacyclovir drug molecule was clearly observed sitting in the central peptide binding site, coordinating a single water molecule (Fig. 1*B*). The valine end of valacyclovir orientates toward the extracellular gate (TMs 1, 2 and 7, 8), which is closed, and the nucleoside part of the drug orientates toward the intracellular gate (TMs 4, 5 and 10, 11), which is open.

**Fig. 2.**
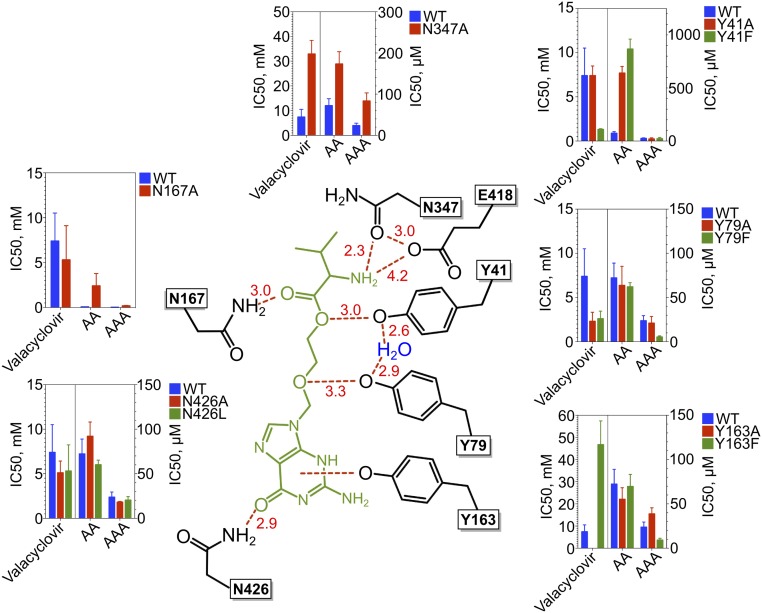
Functional analysis of PepT_Sh_ binding site variants. Schematic of valacyclovir (green) interacting with PepT_Sh_ (black). The contribution of each interacting residue was analyzed using IC_50_ competition assays using three substrates: AlaAla, AlaAlaAla, and valacyclovir are shown. The results are plotted as a bar graph for each variant and compared against WT (blue bars). Interatomic distances (in angstroms) are shown in red.

Valacyclovir makes a number of specific interactions to side chains that are strictly conserved between PepT_Sh_ and both PepT1 and PepT2 (Fig. 2 and *SI Appendix*, Fig. S4). The amino terminus interacts with a conserved glutamate, E418 (E595, human PepT1 numbering) on TM10 and through a hydrogen bond to an asparagine on TM8, N347 (N329). These residues are essential for binding and transport of peptides in both mammalian and bacterial POT family transporters (25, 26). The carbonyl group makes a hydrogen bond to a conserved asparagine, N167 (N171) on TM5, and the ester bond, which links the l-valine to the acyclovir, interacts with tyrosine Y41 (Y40) on TM1. The acyclovir ether group interacts with another conserved tyrosine, Y79 (Y64) on TM2 and the nucleoside portion of the drug makes a distinctive pi-pi stacking interaction with tyrosine Y163 (Y167), also on TM5. Equivalent tyrosine residues play important roles in determining the affinity and transport of peptides in the mammalian proteins (27). The only nonconserved interaction is made between the nucleoside hydroxyl and N426 on TM10, which in PepT1 is a leucine (L630). All these helices are part of the gating architecture within the POT family, which must couple ligand binding to proton movement during transport (28). Of note are the interactions made by the amino terminus of the l-valine scaffold to E418 and N347 and the carbonyl group to N167, as these closely mimic those observed previously in complexes of a related POT family transporter, PepT_St_, to natural peptides (19, 29, 30) and the mammalian homolog PepT1 (31).

### Functional Analysis of Binding Site Residues Contributing to Valacyclovir Recognition.

To further understand the functional importance of these interactions we undertook a detailed functional analysis of the binding site residues (Fig. 2 and *SI Appendix*, Figs. S4 and S5 and Table S2). Previous studies have shown that interactions to the amino terminus of natural peptides are well conserved within the POT family, as are the interactions to the carbonyl oxygen (18). We tested the importance of the N-terminal interactions in PepT_Sh_ by measuring uptake of either N- and C-terminally blocked peptides (*SI Appendix*, Fig. S6). Only the free N-terminal peptide was recognized, consistent with previous reports for the mammalian transporter (26). Further underlining the importance of the N-terminal interactions, a reduction in binding affinity for the N347A variant was also observed (Fig. 2 and *SI Appendix*, Table S2). Mutations of E418 on the other hand resulted in an inactive transporter (*SI Appendix*, Fig. S5), consistent with previous results showing the essential role of the TM10 glutamate in controlling the intracellular gate in response to peptide and proton binding (25, 30).

Prodrugs often contain ester groups, as these confer labile linkages between the active drug molecule and the scaffold, which are easily cleaved once the prodrug has been transported across the membrane (32). An important question is how these functional groups are accommodated within the peptide transporter binding site. The ester linkage interacts through a hydrogen bond with Y41, which plays an important role in peptide recognition and forms part of the conserved ExxERFxYY (33) sequence motif on TM1 (25, 33). However, removing either Y41 or N167, which interacts with the carbonyl group close to the ester linkage, had little effect on the affinity for valacyclovir, whereas a conservative phenylalanine substitution at Y41 resulted in a decrease of the IC_50_ from 7.4 to 1.3 mM. Interestingly, we observe a similar decrease in IC_50_ values for valacyclovir to 2.2 mM for the Y79F variant, which interacts with the ether linkage. These results suggest that while specific interactions to the ester and ether groups in valacyclovir are made, these are not required for prodrug recognition and are merely accommodated within the binding site.

Interestingly however we did observe substantial differences in the IC_50_ values between di- and tripeptides in several of the variants tested (Fig. 2 and *SI Appendix*, Table S2). In particular Y41 appears to play a more important role in dipeptide recognition, as replacement with phenylalanine resulted in an increase in IC_50_ from 72.2 μM to 866 μM, while no effect was observed for trialanine. A similar result was obtained for the Y41A variant. In the Y79F variant, however, we observed the opposite trend, with limited effect on dialanine transport but a positive effect on trialanine recognition, with a reduction in IC_50_ from 23.7 μM to 5.3 μM. The results from the N167A variant also showed a differential effect on di- and trialanine, with the latter being impacted to a far greater degree. These results lend further support to our hypothesis that peptides are accommodated in different positions within the binding site (18) and that this mechanism is shared more widely within the POT family. As we discuss below, a general mechanism for accommodating peptides in different orientations is also supported by the comparison of the crystal structures of valacyclovir and 5-aminolevulinic acid with previous peptide-bound complexes.

Peptide transporters accommodate the chemical diversity of side chain groups within specificity pockets, which contain several conserved tyrosine and polar side chains (18). Unexpectedly the nonprotein purine ring of valacyclovir does not occupy one of these pockets, as previously suggested in an earlier model (34). However, a favorable interaction is observed through a conserved tyrosine, Y163, via pi-pi stacking and with a nonconserved asparagine, N426, through the carbonyl group of valacyclovir. Y163 (Y167) forms part of the PTR2_2 signature motif in the SLC15 family and plays an important role in peptide recognition in human PepT1 (16, 17). With reference to valacyclovir we can extend this function to reveal a key role for tyrosine 163 in accommodating the purine ring. Removal of Y163 abrogated valacyclovir recognition, underscoring its importance. The more conservative phenylalanine substitution also had a negative effect on valacyclovir recognition, increasing the IC_50_ from 7.4 mM to 40 mM. This highlights the importance of the pi-cation interaction between the purine ring and the phenolic hydroxyl group. Removal of N426 again resulted in an improved affinity for valacyclovir, in line with previous results for Y41 and Y79, whereas replacing the side chain with leucine, which is found in the human transporter, had a negligible effect. It is likely the interaction with N426 is specific for PepT_Sh_ and does not occur in the human transporter.

### Structure of 5-Aminolevulinic Acid Complex.

**Fig. 3.**
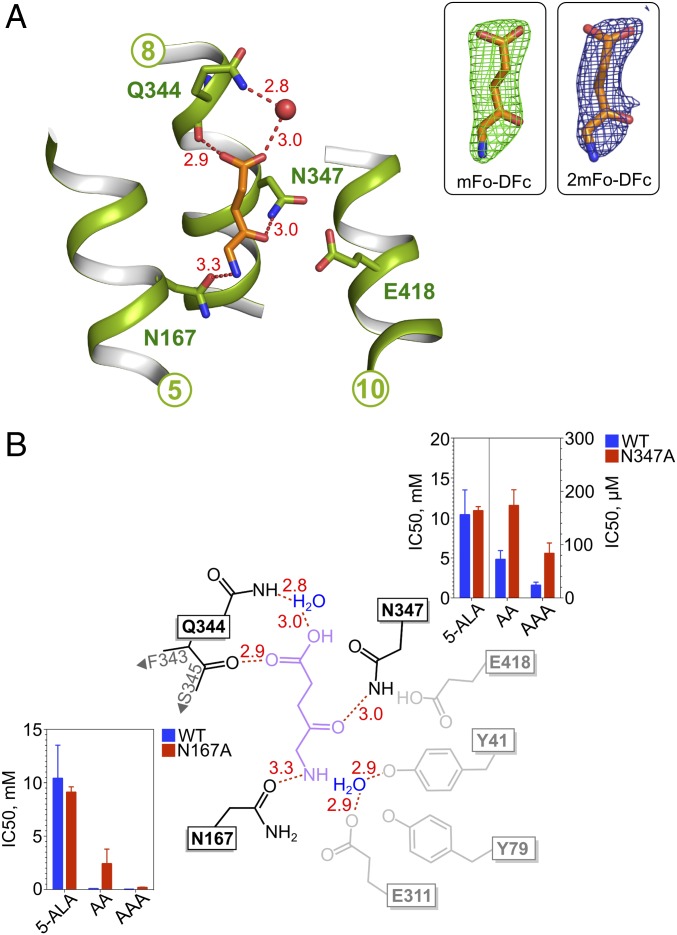
Crystal structure of PepT_Sh_ in complex with 5-aminolevulinic acid. (*A*) Five-aminolevulinic acid (5-ALA, orange sticks) bound to PepT_Sh_ (green). Hydrogen bond interactions are shown as red dashes with distances indicated. Water molecules are shown as red spheres. Key residues involved in binding substrate are shown as green sticks. (*Inset Left*) Experimental *m*Fo-*D*Fc difference electron density (green mesh) observed for 5-aminolevulinic acid, contoured at 3 σ. (*Inset Right*) Final refined 2*m*Fo-*D*Fc electron density map (blue mesh), contoured at 1 σ. (*B*) Schematic of 5-aminolevulinic acid (purple) interacting with PepT_Sh_ (black). Nearby residues that are not interacting with the ligand are indicated in gray. IC_50_ values for the two variants tested are shown as bar charts and compared with WT values. Interatomic distances (in angstroms) are shown in red.

We successfully captured PepT_Sh_ in complex with 5-aminolevulinic acid and determined two structures at 2.5-Å and 2.8-Å resolution, respectively (Fig. 3*A* and *SI Appendix*, Table S1). Five-aminolevulinic acid is an endogenous nonprotein amino acid that forms the first part of the porphyrin synthesis pathway in mammals (35) and is used in the clinic for the photo dynamic detection and treatment of cancer (36). The IC_50_ value for 5-aminolevulinic acid in PepT_Sh_ is 10.4 mM, which is similar to that obtained for valacyclovir of 7.4 mM (*SI Appendix*, Fig. S3*A*), and within the same range observed in PepT1, of 2.1 mM (37). PepT_Sh_ adopts an almost identical inward open state observed previously, with an rmsd of 0.305 Å over 480 C_α_ atoms compared with the valacyclovir structure. However, similar to the valacyclovir structure the B factors and quality of electron density around the cytoplasmic-facing regions of TM10 and -11 clearly indicate increased flexibility in this part of the transporter structure (*SI Appendix*, Fig. S7). This is consistent with previous results obtained for other POT family transporters and lends further support to the C-terminal bundle being more dynamic in this family of MFS proteins (25).

The 5-aminolevulinic acid molecule sits in a similar position as the l-valine scaffold in valacyclovir, adopting a vertical orientation (*SI Appendix*, Fig. S8). However, there are notable differences between how the two molecules interact with the binding site. The C-terminal carboxyl group of 5-aminolevulinic acid faces toward the extracellular gate, making interactions with the backbone carbonyl group of Gln344 (Gln326) and via a water-mediated bridge to the side chain of this same residue. Gln344 forms part of a highly conserved sequence motif at the extracellular part of TM8 in the mammalian peptide transporters (PDQMQ), but only the glutamine is observed in PepT_Sh_ (*SI Appendix*, Fig. S9). The interaction with a backbone carbonyl group and water molecules is similar to the trialanine structure observed in PepT_St_ (19) (PDB: 4D2D), suggesting the vertical mode of binding is associated with lower affinity compared with the more horizontal configuration.

Five-aminolevulinic acid does not contain a peptide bond, having a ketomethylene group instead. This sits in close proximity to E418, in a similar position to the amino terminal group of the l-valine scaffold in valacyclovir. The amino terminal group in contrast sits in a similar position to the carboxyl group of valacyclovir, interacting with N167. Unexpectedly the IC_50_ value for the N167A variant was unchanged (Fig. 3*B*), suggesting this interaction is not essential for binding. It is possible that 5-aminolevulinic acid can form compensatory interactions in the N167A variant or that in the vertical position, the carboxyl group provides compensatory interactions.

Finally, during refinement it became clear that an unusual interaction could be observed within the binding site, wherein an arginine on TM1 (R37) and lysine on TM4 (K137) interact through a shared hydrogen bond (*SI Appendix*, Fig. S10). Arginine 37 forms part of the conserved ExxERFxYY (33) motif on TM1 (*SI Appendix*, Fig. S2), which was previously identified as playing an important role in the proton-coupling mechanism in the POT family (25). Indeed, we previously postulated a role for the conserved arginine in regulating the pK_a_ of the lysine on TM4 (15). However, the current structure provides experimental evidence of a direct interaction between these two side chains. However, we could not discern any significant influence of 5-aminolevulinic acid on the binding site that would cause this interaction to occur, or indeed, of the valacyclovir to break this interaction. Further analysis will be needed to follow up this observation.

## Discussion

Previously determined crystal structures of bacterial peptide transporters in complex with di- and tripeptides have revealed key features of how these ligands are recognized within the POT/SLC15 family (19, 20, 29, 30). To identify commonalities between peptide and prodrug recognition and develop a pharmacophore model for prodrug binding, it is instructive to compare the valacyclovir binding mode with previous peptide-based complex structures. Superposition of the dipeptide-bound crystal structures of the SLC15 transporter from *Streptococcus thermophilus*, PepT_St_ (19, 20) reveal several noticeable commonalities in the binding position of the prodrug and natural peptides (*SI Appendix*, Fig. S11). The l-valine part of valacyclovir makes very similar interactions to the dipeptides l-Ala-l-Phe and l-Ala-l-Gln, while adopting the more vertical orientation observed for the l-Ala-l-Ala-l-Ala tripeptide. The interactions to the amino terminus are well conserved, as is the hydrogen bond to the carbonyl oxygen through N167. Of particular note is the ester linkage part of the valacyclovir prodrug, which closely matches the position of the peptide bond in the dipeptide structures. Indeed, the equivalent tyrosine to Y41 in PepT_St_, which we observe being made to the ester bond in valacyclovir, makes a similar interaction to the amide nitrogen in the peptide bond in the dipeptide complex. It was known previously that although peptide bonds are not strictly required for recognition in PepT1, the presence of a carbonyl group within close proximity to the amino terminus is an important feature of high-affinity ligands (9). It is interesting to note that while neither valacyclovir nor 5-aminolevulinic acid have a peptide bond, they still present hydrogen bond acceptors or donors to both N167 and E418, satisfying this requirement.

Valacyclovir does not contain a terminal carboxy group, which in the dipeptide ligand can be seen making favorable electrostatic interactions to two conserved positively charged side chains in the N-terminal bundle. In valacyclovir we observe the ether bond occupying a similar spatial position as the peptide carboxyl group. It is likely the close placement to arginine 37 (R27) facilitates accommodation of the ether group; however, another interaction to a conserved tyrosine, Y79, is observed.

Of particular interest was the observation that 5-aminolevulinic acid adopted a vertical orientation as opposed to the horizontal one adopted by dipeptides in PepT_St_ (19, 20). It is unclear whether this is due to the presence of the ketomethylene group replacing the peptide bond or the absence of side chains that would be accommodated within the specificity pockets found in the binding sites. A similar vertical orientation was observed for a bound thioalcohol peptide, Cys-Gly-3M3SH, in our previous structure of PepT_Sh_ (22) and a common set of interactions between these three ligands can be discerned, being made to Y41 and N167 (*SI Appendix*, Fig. S12), which are strictly conserved throughput the SLC15 family (*SI Appendix*, Fig. S2). To a lesser extent we also observe interactions to N347, the backbone carbonyl of Q344 and several ordered water molecules. Water molecules have been shown to play an important role in proton movement within peptide transporters (38), and may play a similarly important role in ligand recognition. However, recognition of the unusual Cys-Gly-3M3SH peptide appears to be largely driven through accommodation of the thioalcohol group in an extended hydrophobic pocket, which seems to be an evolutionary adaptation unique to PepT_Sh_ (22).

Our understanding of which scaffolds make optimal candidates for prodrug development is still evolving (6, 13). However, the comparison of the three ligands in PepT_Sh_, and their analysis with respect to peptides bound in PepT_St_, suggests that common points of interaction between the transporter and different ligands exist, which may present a novel route for prodrug scaffold design.

**Fig. 4.**
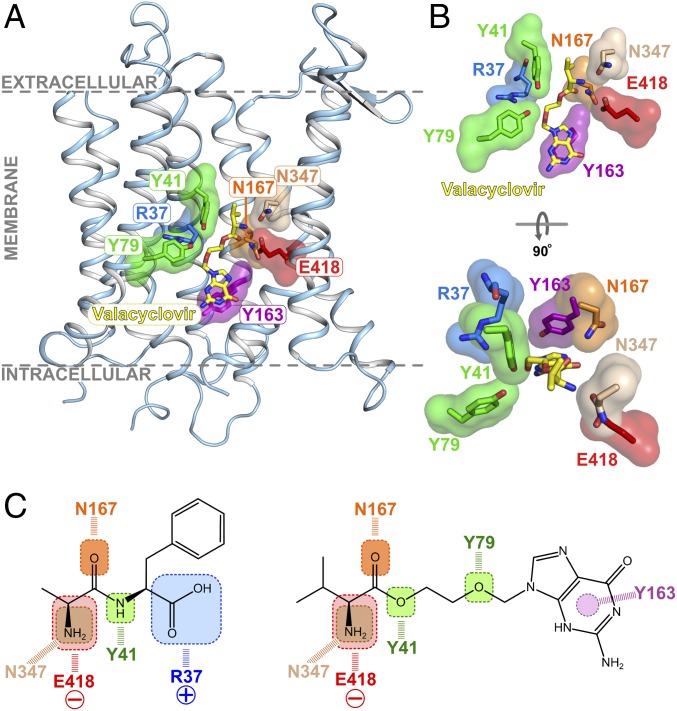
Pharmacophore model for valacyclovir binding to POT family transporters. (*A*) Key interaction sites observed in PepT_Sh_ are shown for valacyclovir in the context of the full transporter structure. (*B*) Closeup view of the PepT_Sh_ binding site accommodating valacyclovir. (*C*) Proposed pharmacophore model indicating the role of conserved SLC15 family binding site residues in recognizing either peptides (AlaPhe) (PDB: 4D2C) or prodrug (valacyclovir).

Taken together these results enable us to propose a structure-based pharmacophore model for valacyclovir binding to peptide transporters (Fig. 4). We are more circumspect regarding a model for 5-aminolevulinic acid, however, as our current mutagenesis data provide less information for this drug. Nevertheless, the structural comparison of valacyclovir with previous dipeptide cocrystal structures suggests that a significant contribution to recognition is made through the amino terminus of the scaffold l-valine, which interacts with N347 and E418. We observe a similar pattern of interactions with the carbonyl group of both the ester bond and the peptide bond to N167. A surprising finding was that while the binding mode of valacyclovir closely replicated that of physiological peptides, the contributions of the interactions to the affinity were noticeably different. Most surprising was that removing the interactions to conserved side chains N167, Y41, and Y79 increased the affinity for valacyclovir, while having a generally negative effect on the transport of di- or trialanine. Importantly, a similar increase in the IC_50_ values for valacylcovir were observed in equivalent variants in the related bacterial peptide transporter from *Shewanella oneidensis*, PepT_So_ (39) (*SI Appendix*, Table S3), lending support to a common mechanism of prodrug recognition within the SLC15 family. Valacyclovir however is much larger than a tripeptide, making the physical constraints of accommodating this molecule more severe. Indeed, the drug appears to occupy a position halfway between the horizontal and vertical poses previously observed in PepT_St_ (19, 20).

A noticeable difference between the valacyclovir and peptide complexes was also observed in the region corresponding to the free carboxyl group. The carboxy terminal group in dipeptides interacts electrostatically with two conserved positively charged side chains in the N-terminal bundle (19). The dipole made between these and the conserved glutamate, E418 on TM10 (E595) helps to orientate smaller peptides in the binding site. The absence of a carboxyl group in valacyclovir results in no interactions being made to the positive cluster in the N-terminal bundle. However, a free carboxyl group is not required for peptide recognition in mammalian PepT1 (26) or PepT_Sh_ (*SI Appendix*, Fig. S6), explaining why this interaction is not strictly required. A further important observation was the role played by Y163 in accommodating the nonpeptidometic purine ring within the binding site. This result supports a broader role for tyrosine side chains in contributing to the drug binding within the human peptide transporters. Additionally, the structural comparison with valacyclovir further develops our previous hypothesis that the promiscuity observed in peptide transporters stems in part from their ability to accommodate ligands in different orientations in the binding site (18). This “multimode binding model” for ligand recognition would also explain why prodrug molecules can be accommodated through interactions with the amino terminal group on the scaffold and steric complementarity between the ester linkage and peptide backbone.

## Concluding Remarks

The development of prodrugs has progressed with the aim of improving drug pharmacokinetics by overcoming various barriers that reduce clinical efficacy, such as poor oral bioavailability or cellular toxicity due to adverse drug–drug interactions (1). Carrier-mediated prodrug design has been successfully employed to overcome these challenges (6); however, prodrug design remains difficult owing to the lack of a generally applicable strategy that can be broadly applied. The unusual promiscuity of PepT1 and PepT2 make them ideal targets for prodrug development (7). The current crystal structures and the associated pharmacophore model for valacyclovir and 5-aminolevulinic acid recognition by PepT_Sh_ provides an important advance in our attempts to rationalize and exploit PepT1 and PepT2 more widely in carrier-mediated drug transport.

## Methods

### Crystallization and Structure Determination.

Purified PepT_Sh_ protein was produced as previously described (22) and incubated with either 40 mM valacyclovir hydrochloride (Sigma-Aldrich) or 5-aminolevulinic acid and left for 4 h at 4 °C before crystallization. The protein-laden mesophase was prepared by homogenizing monoolein (Sigma) and 10 mg⋅mL^−1^ protein solution in a 60:40 ratio by weight using a dual syringe mixing device at 20 °C. Crystallization was carried out at 4 °C in 96-well glass sandwich plates against 26–27% (vol/vol) PEG 200, 220 mM (NH4)_2_HPO_4_, and 110 mM sodium citrate (pH 5.0). Crystals grew within 2 to 3 d. Wells were opened using a tungsten-carbide glasscutter and the crystals were harvested using 100-µm micromounts (MiTeGen). Crystals were cryocooled directly in liquid nitrogen. Data were collected at beamline I24 (Diamond Light Source) and ID23eh2 (European Synchrotron Radiation Facility).

### Model Building and Refinement.

The structure was phased by molecular replacement using the previously resolved PepT_Sh_ crystal structure, PDB: 6EXS. The model was built into the resulting electron density maps followed by refinement in Phenix (40). Figures were prepared using PyMOL (Schrödinger, LLC).

### Fluorescence-Based Transport and Competition Assays.

Transport assays were carried out as described previously (22), employing a Cary Eclipse fluorescence spectrophotometer (Agilent Technologies) to measure the change in fluorescence of the pH-sensitive dye pyranine. Dual fluorescence excitation was set to 460/415 nm with emission at 510 nm. Transport was initiated following addition of 1 μM valinomycin. Competition assays were performed at 30 °C, with samples taken at specified time points. Proteoliposomes were immediately filtered onto a 0.22-μm cellulose filter (Merck Millipore) using a vacuum manifold and washed twice with 2 mL cold H_2_O. The amount of peptide transported into the liposomes was calculated based on the specific activity for each peptide. Experiments were performed a minimal of four times to generate an overall mean and SD and the resulting data were analyzed using Prism 7.0 (GraphPad Software).

### Data Availability.

Atomic coordinates have been deposited in the Protein Data Bank (PDB) under accession numbers 6GZ9 (valacyclovir complex) and 6H7U and 6HZP (5-aminolevulinic acid complexes).

## Supplementary Material

Supplementary File
