# Supplementary material for: Structural basis for prodrug recognition by the SLC15 family of proton-coupled peptide transporters
Source: Proc Natl Acad Sci U S A. 2019 Jan 2;116(3):804–9. doi: 10.1073/pnas.1813715116 (PMC6338836; doi:10.1073/pnas.1813715116)
Supplement: Supplementary File [file pnas.1813715116.sapp.pdf]

Supplementary Material: Figures 1-12 & Tables 1-3.

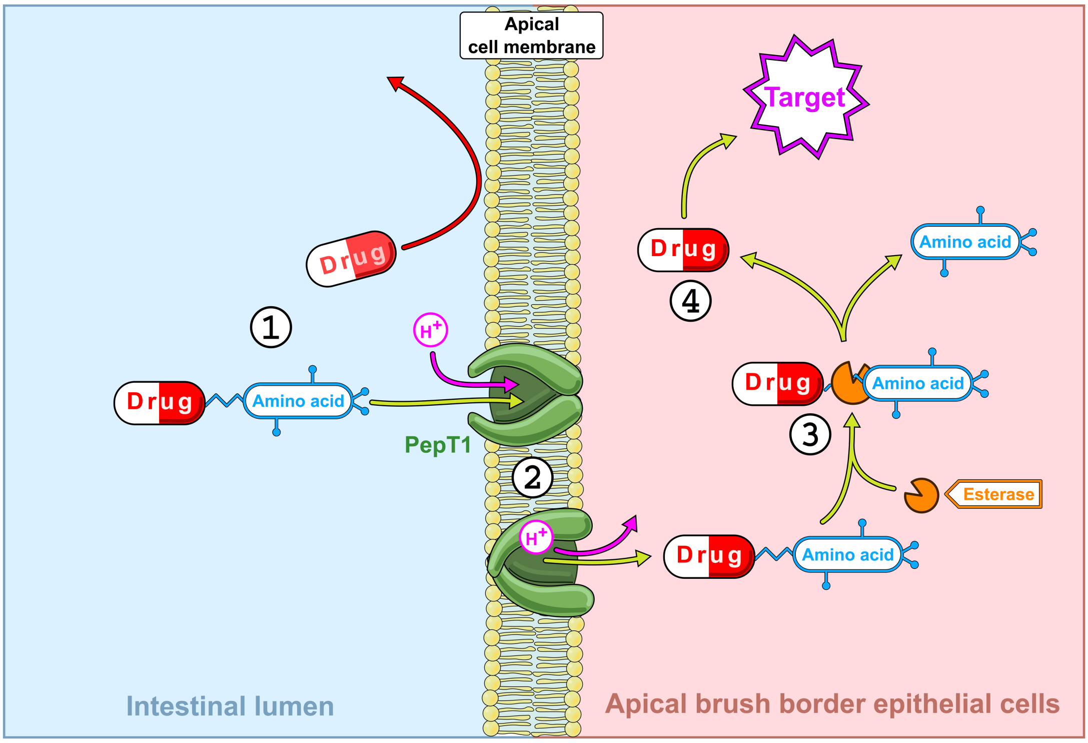

Supplementary Figure 1. Schematic of prodrug mediated transport via human hPEPT1 & PepT2. The unmodified hydrophilic drug is membrane impermeable, unable to access the cell (1). The prodrug, an amino acid ester derivative of the drug, is however recognised by hPEPT1 and actively transported across the apical cell membrane into the cell (2). After transport the prodrug is hydrolysed and converted into its pharmacologically active form through the action of non-specific esterases in the cytoplasm (3), yielding the active parent drug inside the cell (4).

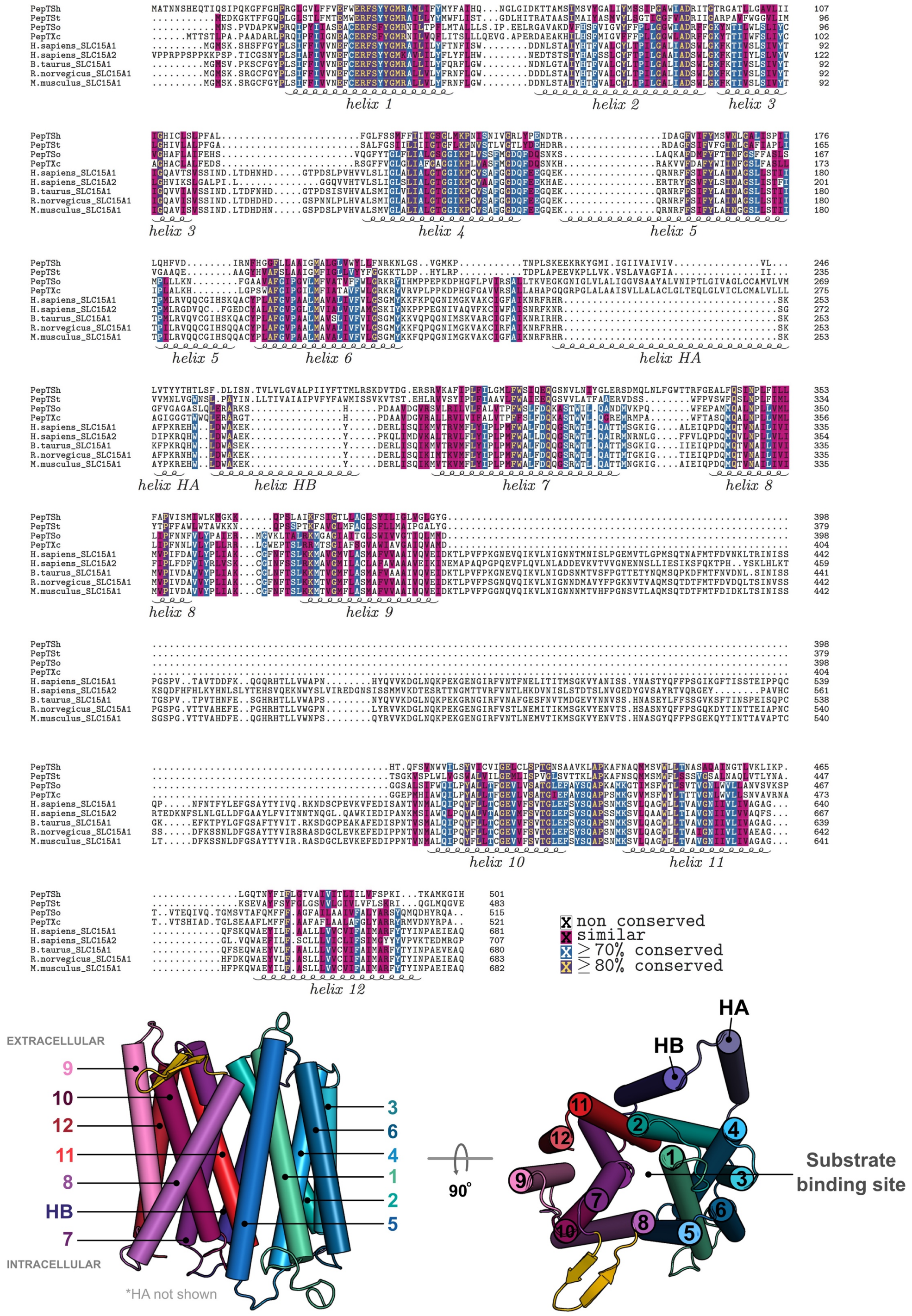

Supplementary Figure 2. Sequence alignment of PepT$_{Sh}$ with bacterial and human POT homologues. The sequence alignment was made with Clustal Omega on default settings. Highly conserved (>80%) amino acids are highlighted in purple. Similar amino acids are coloured in magenta. The locations of transmembrane helices within the sequences are highlighted. The crystal structure of PepT$_{Sh}$ (PDB: 6EXS) with TM helices highlighted is shown at the bottom. Sequence alignments were coloured using TeXShade.

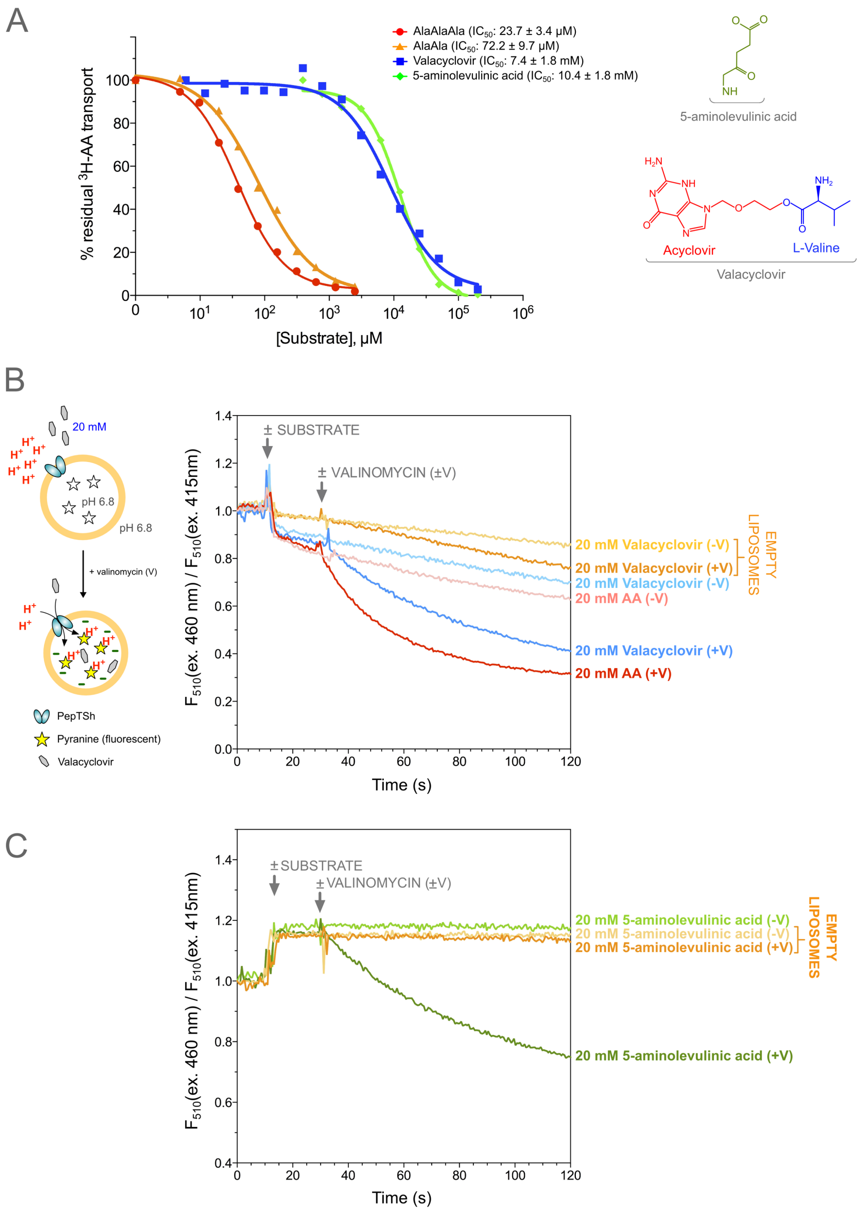

Supplementary Figure 3. Valacyclovir and 5-aminolevulinic transport by PepT$_{Sh}$. (A) IC_{50} competition curves for dipeptide (AlaAla), tripeptide (AlaAlaAla), valacyclovir and 5-aminolevulinic acid in PepT$_{Sh}$. Right panel: Chemical structures of 5-aminolevulinic acid (green) and valacyclovir (L-valine scaffold shown in blue, acyclovir in red). (B) Measurement of valacyclovir transport using a pH reporter assay. PepT$_{Sh}$ was reconstituted into liposomes loaded with pyranine and potassium ions. The external solution contains either peptide, AlaAla (red) or valacyclovir (dark blue) and low potassium. On addition of valinomycin (+V) a membrane potential (negative inside) is generated, driving transport. (C) Measurement of 5-aminolevulinic acid transport as in (B).

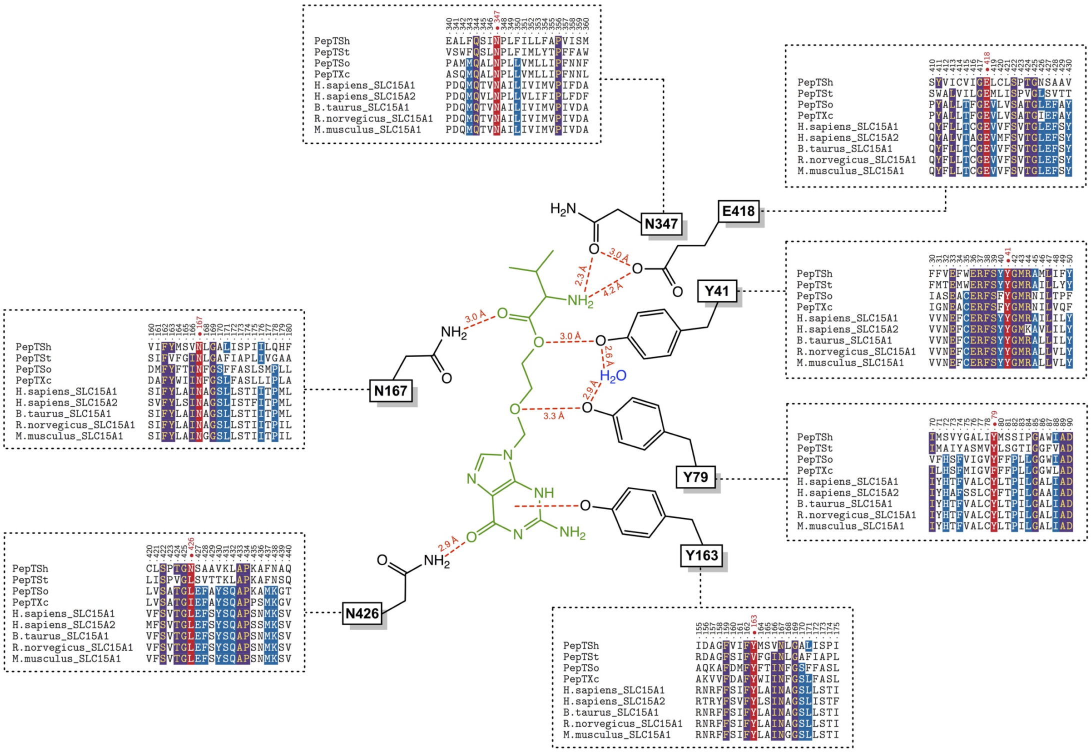

Supplementary Figure 4. Sequence alignments showing conservation of binding site residues within the POT family. $PepT_{Sh}$ residues participating in binding valacyclovir are highlighted in red, and aligned against other bacterial and mammalian homologues. Sequence conservation in homologs over 75% are coloured blue. Sequence conservation >90% are coloured purple.

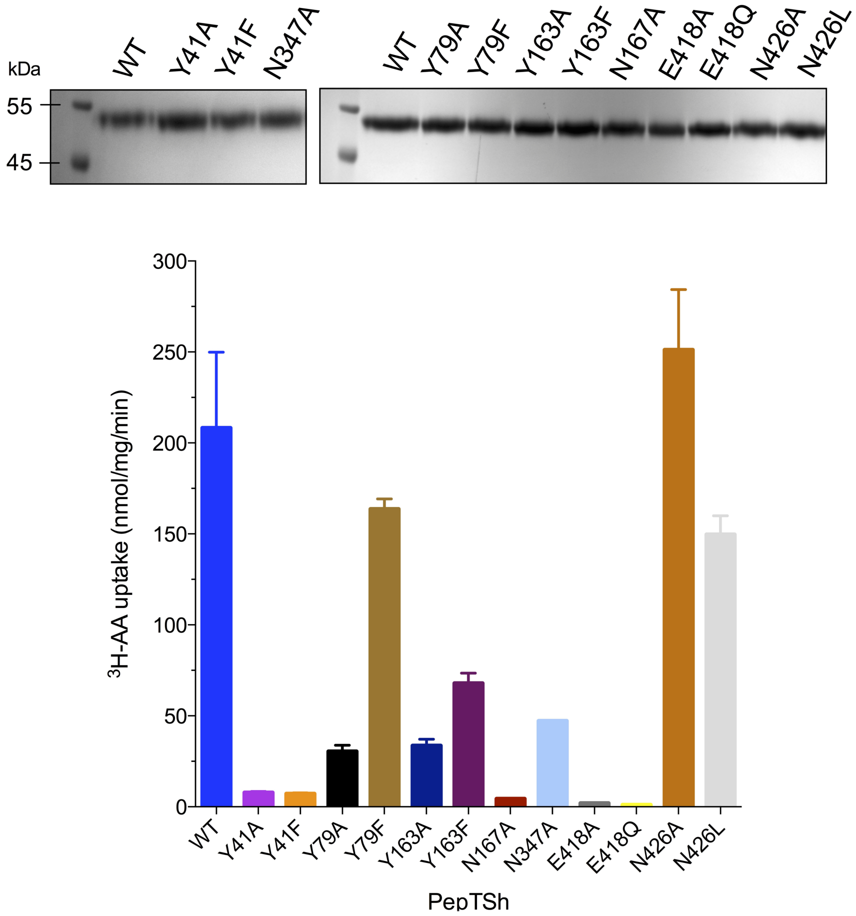

Supplementary Figure 5. Functional characterisation of binding site residues. Bar chart comparing AlaAla uptake in proteoliposomes for $PepT_{Sh}$ variants compared to WT levels. Inset: SDS-PAGE analysis of the reconstituted proteins used in the transport assays.

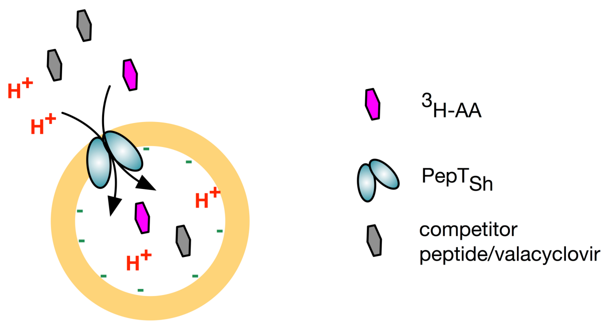

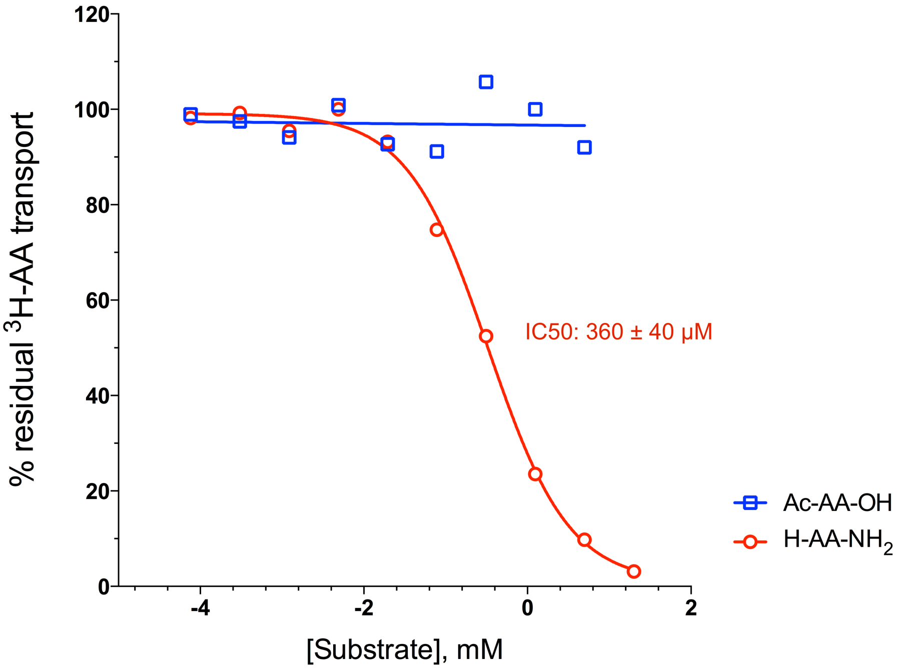

Supplementary Figure 6. The N-terminus of peptide ligands is essential for recognition in PepT$_{Sh}$. IC$_{50}$ competition curve showing the importance of N-terminal recognition within POT family transporters. Only the peptide with a free N-terminus (H-AA-NH$_2$, red) can compete with AlaAla, whereas the N-terminally blocked peptide (Ac-AA-OH, blue) can no longer bind the transporter.

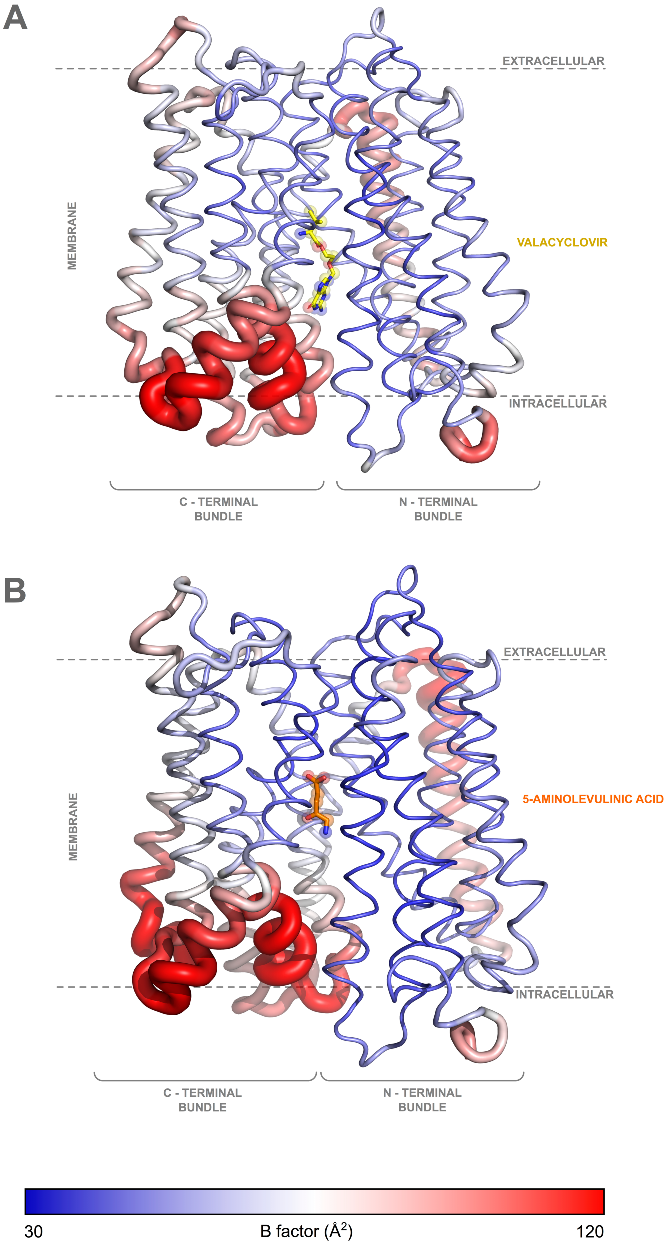

Supplementary Figure 7. Temperature factor analysis of PepT$_{Sh}$ bound to valacyclovir and 5-aminolevulinic acid. The C-terminal domain of PepT$_{Sh}$ has noticeably higher B factors in both (A) valacyclovir and (B) 5-aminolevulinic acid between 90-120 Å^2, indicating increased flexibility in this region of the transporter.

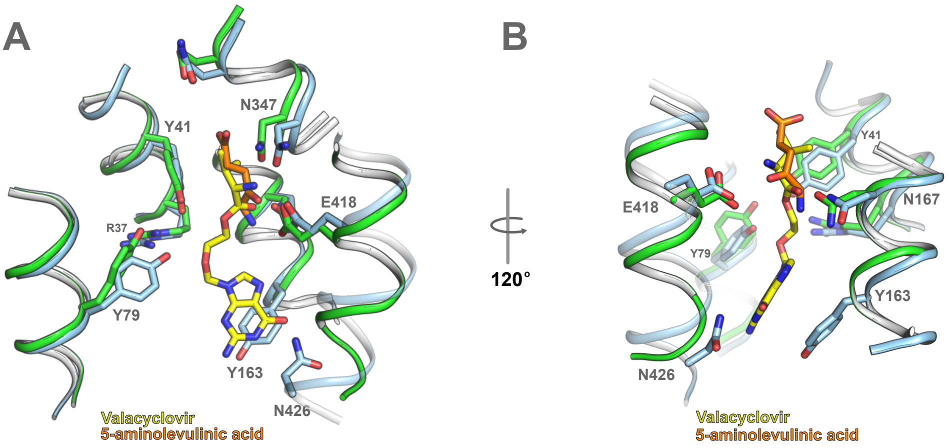

Supplementary Figure 8. Structural comparison between valacyclovir and 5-aminolevulinic acid binding positions. (A) The crystal structure of the valacylcovir complex (blue) and 5-aminolevulinic acid (green) is shown. The 5-aminolevulinic acid molecule sits in a similar position to the L-valine scaffold in valacyclovir. However, the orientation of the N- and C-terminal groups is different, with 5-aminolevulinic acid adopting the opposite orientation in the binding site. (B) View rotated 90°.

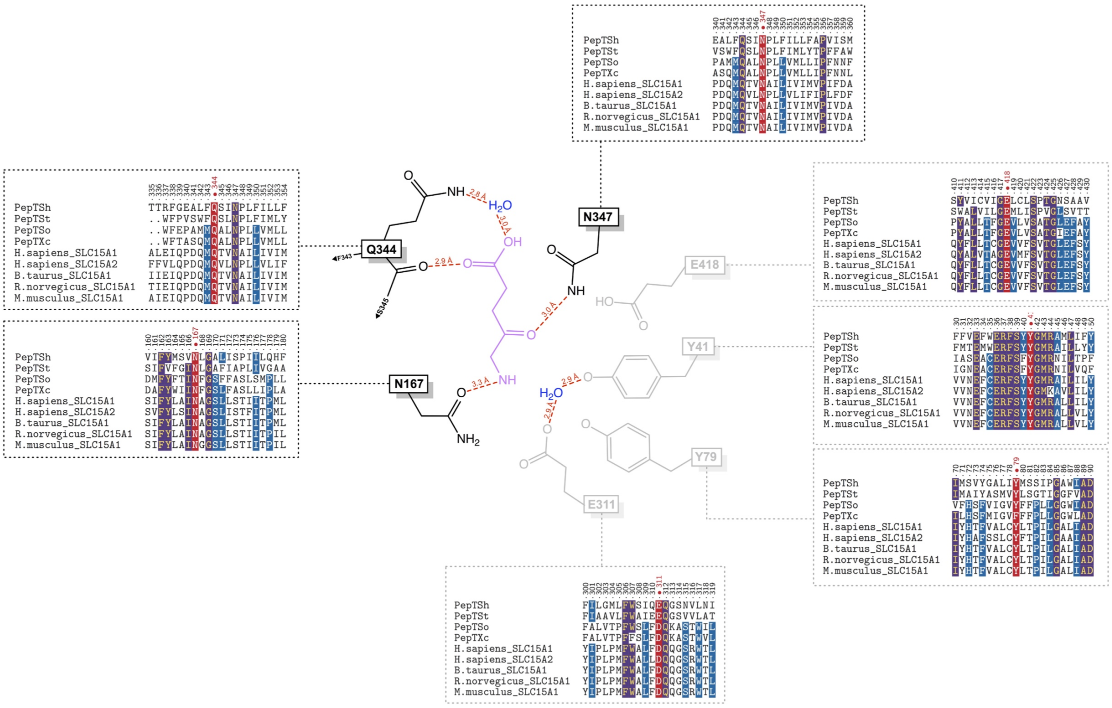

Supplementary Figure 9. Sequence analysis showing conservation of key residues interacting with 5-aminolevulinic acid. Schematic of the key interaction sites in PepT$_{Sh}$ are shown in black, with nearby residues in grey. Residues shown are highlighted in red. The sequence conservation is shown for each residue across bacterial and mammalian SLC15 family members. Sequence conservation in homologs over 75% are coloured blue. Sequence conservation >90% are coloured purple.

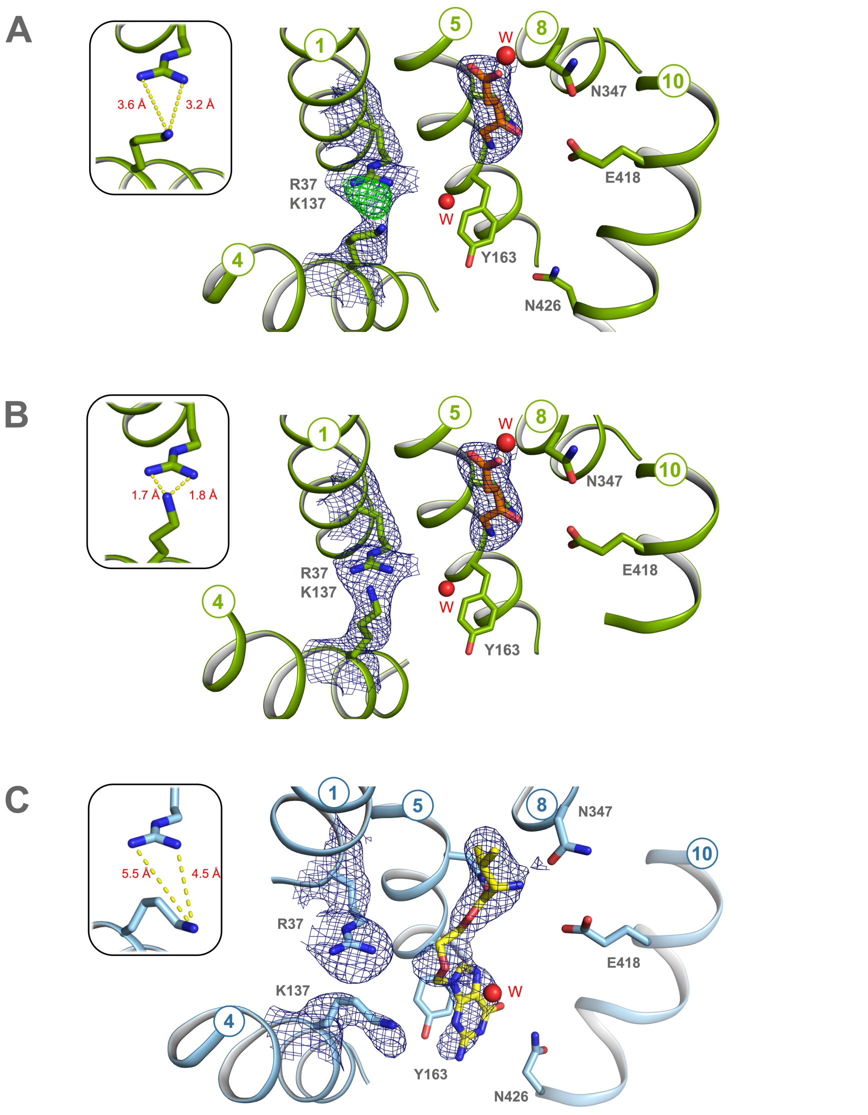

Supplementary Figure 10. Analysis of interaction between Arg37 and Lys137 in PepT$_{Sh}$. (A) Unmodeled interaction between R37 and K137 in a 5-aminolevulinic acid structure (PDB: 6H7U) compared to the final refined density (B) following the introduction of a LINK card in the PDB header. The same interaction is also present in a second complex with 5-aminolevulinic acid (PDB:6HZP) (SI Table 1). It is clear from the difference electron density in both structures that K137 interacts with R37, most likely through a hydrogen bond, consistent with their role in proton coupling in the POT family. (C) Equivalent view in the valacyclovir structure (PDB: 6GZ9) showing a broken interaction. Inset – close up view of the side chains showing final refined distances.

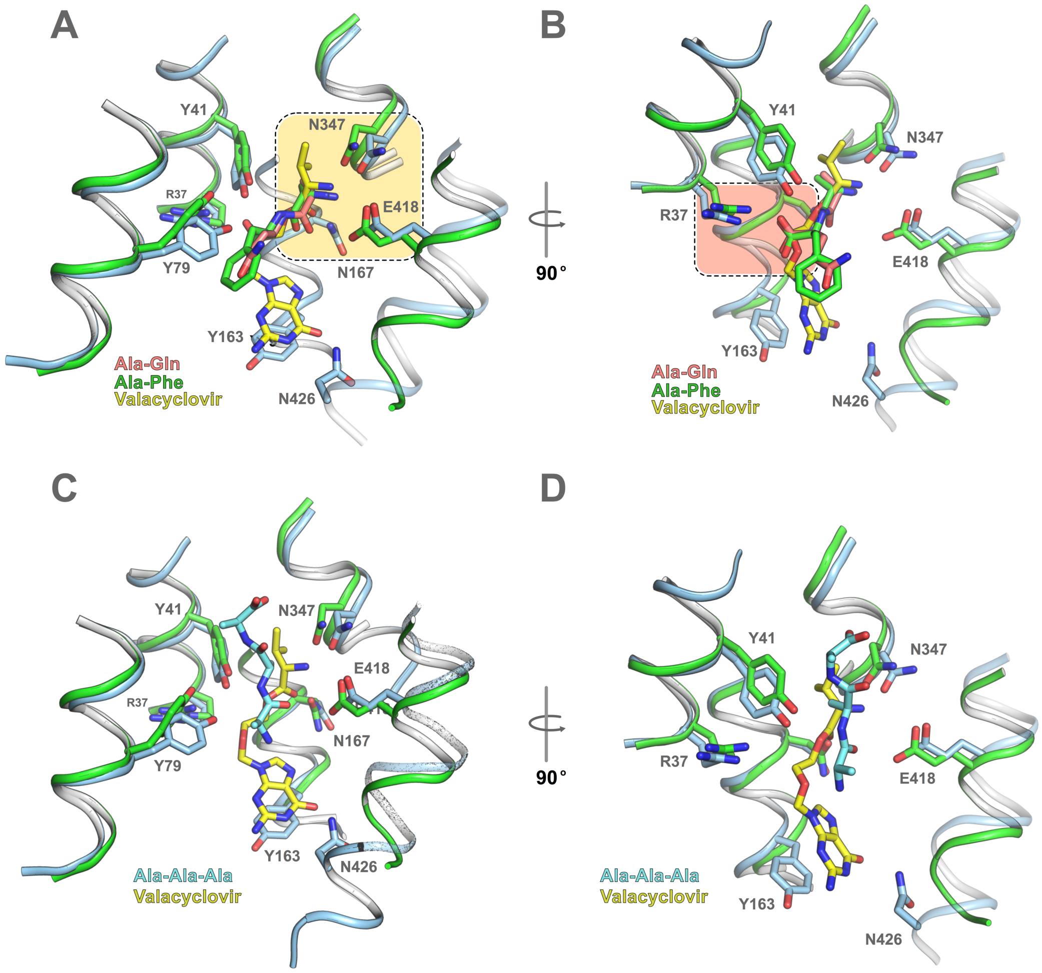

Supplementary Figure 11. Structural comparison of binding sites in PepT$_{Sh}$ and PepT$_{St}$. A closer view of the substrate binding site in PepT$_{Sh}$ (PDB: 6GZ9) (blue) overlaid onto PepT$_{St}$ (PDB:4D2C) (green). Valacyclovir is shown as yellow sticks, AlaPhe and AlaGln (PDB: 5OXK) are shown as green and pink sticks respectively. (A) The yellow square highlights the area where the amino terminus of valacyclovir, AlaPhe, and AlaGln interact with a conserved glutamate (E418) and a conserved asparagine (N347). (B) View rotated 90°. The red square illustrates that the carboxy terminus from the dipeptide ligands interacting with arginine (R37). In valacyclovir it is likely the ether group compensates for the absence of a carboxy terminus. (C) Similar to the binding position of a previously captured tri-peptide ligand AlaAlaAla in PepT$_{St}$ (PDB: 4D2D) the L-valine in valacyclovir adopts a vertical orientation. (D) View rotated 90°

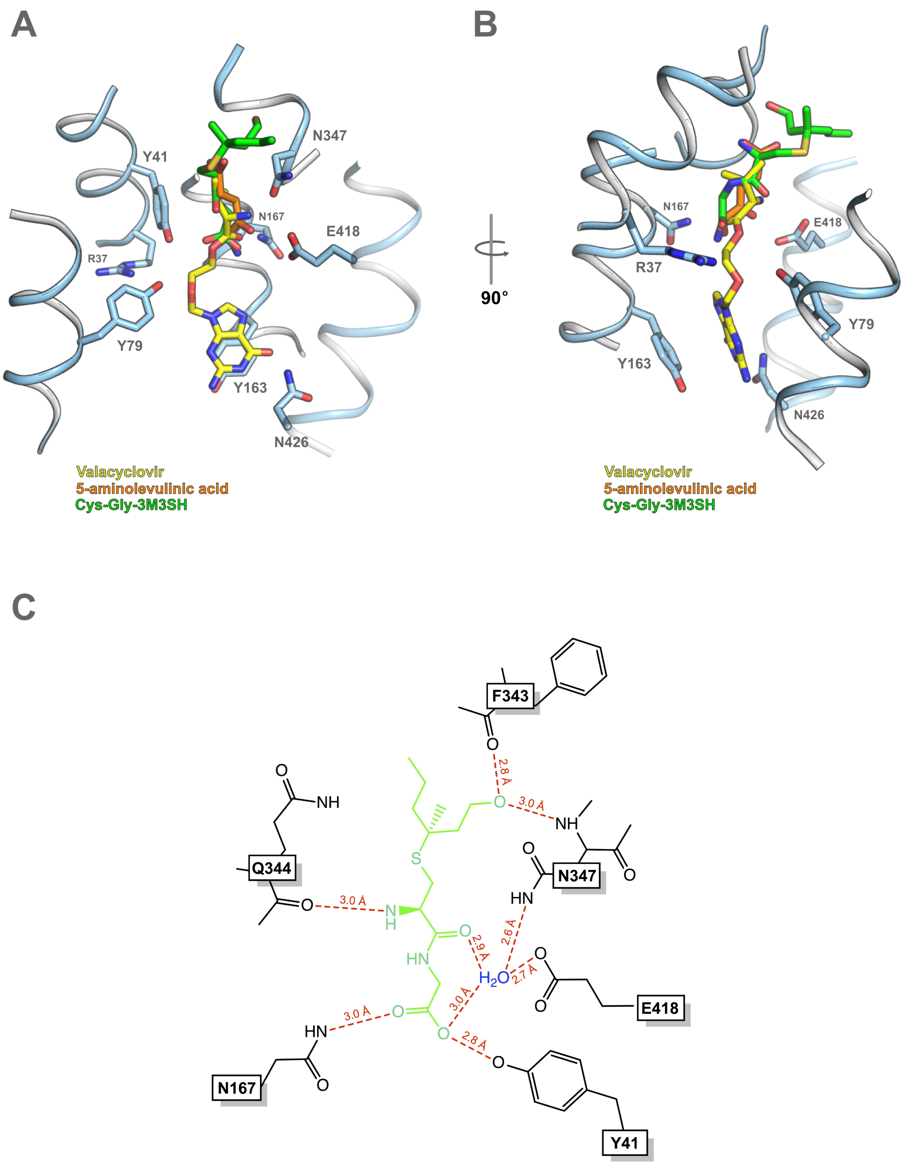

Supplementary Figure 12. Structural comparison between the Cys-Gly-3M3SH and drug bound structures of $\mathrm{PepT_{Sh}}$. (A) Close up view of the valacyclovir complex (PDB: 6GZ9) (yellow) overlaid with both 5-aminolevulinic acid (PDB: 6HZP) (orange) and Cys-Gly-3M3SH (PDB: 6EXS) (green) structures. (B) View rotated 90° with respect to (A). (C) Schematic of the binding site in $\mathrm{PepT_{Sh}}$ showing the interactions made to the Cys-Gly-3M3SH peptide.

Supplementary Table 1. Data collection and statistics.

	6GZ9 PepT$_{Sh}$ Valacyclovir	67HU PepT$_{Sh}$ 5-aminolevulinic acid	6HZP PepT$_{Sh}$ 5-aminolevulinic acid
**Data collection**			
Space group	P 1 21 1	P 1 21 1	P 1 21 1
Cell dimensions			
a, b, c (Å)	60.1, 56.6, 99.2	60.5, 57.0, 100	60.0, 57.1, 99.50
α, β, γ (°)	90.0, 104.6, 90.0	90.0, 104.8, 90.0	90.0, 104.9, 90.0
Wavelength (Å)	0.9686	0.8729	0.8729
Resolution (Å)[a]	58.2 – 3.1	40.3 – 2.8	49.0 – 2.5
CC1/2 (%)	0.987 (0.57)	0.992 (0.791)	0.998 (0.635)
R_{merge}	0.261 (1.775)	0.1854 (1.148)	0.137 (1.274)
R_{pim}	0.115 (0.745)	0.0796 (0.488)	0.163 (0.807)
$I/\sigma I$	5.65 (1.19)	7.74 (1.74)	11.1 (1.15)
Completeness (%)	98.67 (99.92)	98.20 (99.69)	99.6 (99.9)
Redundancy	6.3 (6.6)	6.6 (6.5)	6.6 (6.7)
**Refinement**			
Resolution (Å)	58.2 – 3.1	40.3 – 2.8	49.0 – 2.5
Number of reflections	11907	10553	22705
R_{work} / R_{free}	0.244/0.305	0.293/0.320	0.219/0.239
B-factors (Å^2)			
Protein	69.1	71.5	65.2
Ligand	84.8	66.7	93.3
Solvent	55.6	42.8	53.0
R.M.S deviations			
Bond lengths (Å)	0.009	0.014	0.013
Bond angles (°)	1.17	1.63	1.41
Ramachandran statistics	90.12/0.82	96.91/0.00	98.14/0.00
Favoured/outliers (%)			
Molprobity score	96th percentile	100th percentile	100th percentile

[a] Highest resolution shell shown in parenthesis.

Supplementary Table 2

IC_{50} values measured for WT and variants of $PepT_{Sh}$.

$PepT_{Sh}$ variant	Valacyclovir (IC_{50})	Ala-Ala (IC_{50})	Ala-Ala-Ala (IC_{50})	% WT activity
WT	7.4 ± 1.8 mM	72.2 ± 9.7 μM	23.7 ± 3.4 μM	100
Y41A	7.4 ± 0.6 mM	640.4 ± 36.4 μM	18.5 ± 6.7 μM	4
Y41F	1.3 ± 0.1 mM	866.5 ± 54.6 μM	21.4 ± 7.1 μM	4
Y79A	2.3 ± 0.6 mM	63.6 ± 12.6 μM	21.4 ± 4.4 μM	15
Y79F	2.6 ± 0.5 mM	62.0 ± 2.8 μM	5.3 ± 0.8 μM	78
Y163A	N.D.	55.2 ± 7.5 μM	38.6 ± 7.0 μM	16
Y163F	46.7 ± 6.2 mM	69.6 ± 7.9 μM	9.1 ± 2.1 μM	33
N167A	5.3 ± 2.2 mM	2.4 ± 0.4 mM	182.8 ± 26.9 μM	2
N347A	32.9 ± 3.2 mM	173.2 ± 17.3 μM	83.3 ± 11.5 μM	23
N426A	5.1 ± 0.8 mM	92.0 ± 9.3 μM	18.1 ± 0.5 μM	120
N426L	5.3 ± 1.7 mM	60.1 ± 3.0 μM	20.4 ± 2.3 μM	72

N.D. Result was ambiguous due to inability to transport.

Supplementary Table 3

IC_{50} values measured for WT and variants of $PepT_{So}$.

**$PepT_{So}$ variant** ($PepT_{Sh}$ equivalent variant)	**Valacyclovir** (IC_{50})	**Ala-Ala** (IC_{50})	**Ala-Ala-Ala** (IC_{50})	**WT activity (%)**
WT	4.5 ± 0.7 mM	115.2 ± 0.8 μM	510.2 ± 71.6 μM	100
Y29F$^{(Y41F)}$	2.7 ± 0.1 mM	378.4 ± 124.7μM	207.7 ± 8.9 μM	23
Y68F$^{(Y79F)}$	3.4 ± 1.1 mM	139.2 ± 30.4 μM	927.5 ± 32.7 μM	72
